# Shoc2 Is Targeted to Late Endosomes and Required for Erk1/2 Activation in EGF-Stimulated Cells

**DOI:** 10.1371/journal.pone.0036469

**Published:** 2012-05-14

**Authors:** Emilia Galperin, Lina Abdelmoti, Alexander Sorkin

**Affiliations:** 1 Department of Molecular and Cellular Biochemistry, College of Medicine, University of Kentucky, Lexington, Kentucky, United States of America; 2 Department of Cell Biology, University of Pittsburgh, School of Medicine, Pittsburgh, Pennsylvania, United States of America; University of North Carolina, United States of America

## Abstract

Shoc2 is the putative scaffold protein that interacts with RAS and RAF, and positively regulates signaling to extracellular signal-regulated protein kinases 1 and 2 (ERK1/2). To elucidate the mechanism by which Shoc2 regulates ERK1/2 activation by the epidermal growth factor (EGF) receptor (EGFR), we studied subcellular localization of Shoc2. Upon EGFR activation, endogenous Shoc2 and red fluorescent protein tagged Shoc2 were translocated from the cytosol to a subset of late endosomes containing Rab7. The endosomal recruitment of Shoc2 was blocked by overexpression of a GDP-bound H-RAS (N17S) mutant and RNAi knockdown of clathrin, suggesting the requirement of RAS activity and clathrin-dependent endocytosis. RNAi depletion of Shoc2 strongly inhibited activation of ERK1/2 by low, physiological EGF concentrations, which was rescued by expression of wild-type recombinant Shoc2. In contrast, the Shoc2 (S2G) mutant, that is myristoylated and found in patients with the Noonan-like syndrome, did not rescue ERK1/2 activation in Shoc2-depleted cells. Shoc2 (S2G) was not located in late endosomes but was present on the plasma membrane and early endosomes. These data suggest that targeting of Shoc2 to late endosomes may facilitate EGFR-induced ERK activation under physiological conditions of cell stimulation by EGF, and therefore, may be involved in the spatiotemporal regulation of signaling through the RAS-RAF module.

## Introduction

Organization of signaling modules in macromolecular complexes by scaffold proteins has an important role in regulating intracellular signaling in time and space and defining its input/output strength [1], [2], [3]. Scaffold proteins tether signaling components and localize them to specific areas of the cell, providing microenvironments where the concentration of interacting partners is greatly increased [4]. In addition, scaffolds regulate signal transduction by coordinating positive and negative feedback signals, and by shielding correct signaling proteins from irrelevant stimuli [1].

The signaling cascade leading to activation of mitogen-activated protein kinase/extracellular stimulus-regulated kinase 1 and 2 (MAPK/ERK1/2) is an intricate system that is regulated at multiple cellular sites [5]. The ERK1/2 activation cascade is initiated by various extracellular stimuli leading to GTP loading of RAS, recruitment of the RAF kinase to GTP-RAS, phosphorylation and activation of the MAPK kinase (MEK1 and 2) by RAF, and finally activating phosphorylation of ERK1/2 by MEK1/2 [6]. The outcome of ERK1/2 activation ultimately depends on the set of substrates that ERK1/2 phosphorylates at specific cellular locations. In many instances, this complex pathway is regulated by a number of accessory proteins and, in particular, scaffold proteins [7]. Scaffolds bind the components of the ERK1/2 signaling cascade, bring them together and target multi-protein signaling modules to different cellular locations, thus enhancing phosphorylation of specific substrates [8]. Overexpression of scaffold proteins often results in the sequestration of their interaction partners to non-specific complexes, which disturbs the ERK activation process and its regulation [9].

Several scaffold proteins have been shown to localize components of the ERK1/2 cascade to specific cellular locations. Kinase suppressor of RAS (KSR) is the best-studied scaffold of the ERK1/2 pathway that is conserved from *C. elegans* to humans [10]. KSR forms multi-component complexes at the plasma membrane, bringing together RAF, MEK and ERK [11]. MEK Partner 1 (MP-1) was identified originally as a MEK1 binding protein and later was reported to have scaffolding properties [12], [13]. MP-1 interacts specifically with MEK1 and ERK1, enhances their interaction and recruits these complexes to late endosomes. MP-1 complex is anchored to the endosomal membrane by means of binding to the late-endosomal resident proteins, p14 and p18 [14]. Loss of individual components of the MP-1/p14/p18 complex reduces duration of the ERK1/2 activity, thus implicating late endosomes as an important platform for the ERK1/2 signaling [15]. Furthermore, endoplasmic reticulum and Golgi apparatus serve as platforms for the scaffold complex formed by BIT1 (Bcl-2 inhibitor of transcription) [16]. It has been suggested that this complex provides a negative feedback to ERK1/2 signaling and impacts cell adaptation to stress and resistance to death [16].

In the present study, we analyzed how a putative scaffold protein Shoc2 contributes to the regulation of the ERK1/2 pathway. This leucine-repeat rich protein was first identified in *C. elegans* (named SOC-2/SUR-8), and demonstrated to interact differentially with various RAS proteins and positively regulate RAS-mediated signaling [17]. The human homolog of SOC2/SUR-8 (Shoc2) was also shown to facilitate ERK1/2 signaling and interact with RAS and RAF, forming a ternary complex with these two proteins [17], [18]. Moreover, it has been recently demonstrated that Shoc2 regulates the rate of RAS-RAF interaction [19], [20]. In addition, Shoc2 was proposed to regulate ERK1/2 activity as part of a holoenzyme comprised of the catalytic subunit of protein phosphatase PP1C and Shoc2 [21]. PP1C is recruited to RAF-1 via Shoc2 where it dephosphorylates an inhibitory residue Ser259 allowing for activation of RAF-1 by phosphorylation. Furthermore, Shoc2 was shown to modulate Ca^2+^- and calmodulin-dependent regulation of RAF-1 activation [19], [20]. Recent studies reported that the S2G mutation in Shoc2 is associated with Noonan-like (NL) syndrome, a genetically inherited disease manifested in loose anagen hair [22]. The latter study demonstrated that this mutation results in Shoc2 N-myristoylation and, therefore, mistargeting of Shoc2 to the plasma membrane.

To elucidate the mechanisms of regulation of the ERK1/2 activation cascade by Shoc2, we analyzed subcellular localization of Shoc2 and found that upon stimulation of cells with epidermal growth factor (EGF), Shoc2 rapidly accumulated on a subset of late endosomes. RNAi knockdown and complementation experiments demonstrated that Shoc2 is required for the efficient ERK1/2 activation by EGF, and suggested that translocation to late endosomes may be a part of the regulatory mechanism which underlies Shoc2-dependent ERK1/2 activation.

## Results

### EGFR Activation Triggers Recruitment of Shoc2 to Endosomes

To elucidate the mechanisms by which Shoc2 regulates the EGFR-RAS-ERK1/2 signaling cascade, sub-cellular localization of Shoc2 was studied using fluorescence microscopy. For studies in living cells, tagRFP (tRFP)-fusion protein of Shoc2 was prepared, in which tRFP was attached to the C- terminus of Shoc2. The ability of Shoc2-tRFP to associate with its interaction partner, RAS, was demonstrated by co-immunoprecipitation of Shoc2-tRFP with HA-tagged H-RAS and M-RAS expressed in 293FT cells (Figure S1). Shoc2-tRFP interaction with RAS proteins was abolished by a D175N mutation, previously shown to interfere with RAS binding [17], [18] (Figure S1). Therefore, based on the demonstrated functionality of Shoc2-tRFP, we used this fusion protein in subsequent experiments.

**Figure 1:**
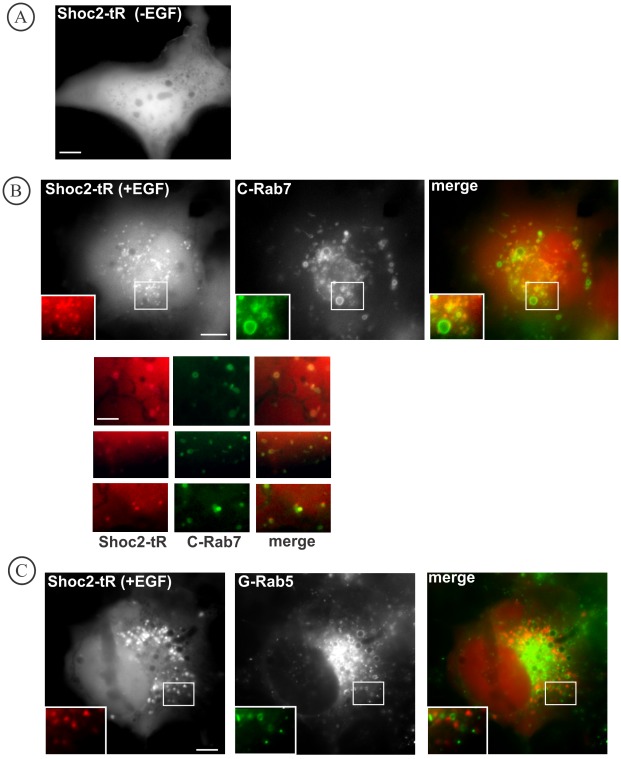
Localization of Shoc2-tRFP and characterization of Shoc2-tRFP containing compartments. **A**, Cos1 cells were transfected with Shoc2-tRFP and imaged live before EGF treatment. Shoc2-tRFP is located in the cytosol and nucleus. Scale bar, 10 µm. **B**, Shoc2-tRFP and CFP-Rab7 were transiently expressed in Cos1 cells. Cells were serum-starved for 16 h and then treated with 10 ng/ml EGF for 12 min at 37°C. Insets show high magnification images of the regions of the cell indicated by white rectangles, Scale bar, 10 µm. A panel below shows multiple high-magnification images with examples of co-localization of Shoc2-tRFP with CFP-Rab7. Scale bar, 5 µm**. C**, Shoc2-tRFP and GFP-Rab5 were transiently expressed in Cos1 cells. Cells were serum-starved for 16 hr. Cells were then treated with 10 ng/ml EGF for 12 min at 37°C. Insets show high magnification images of the regions of the cell indicated by white rectangles, Scale bar, 10 µm.

Analysis of the subcellular localization of the transiently expressed Shoc2-tRFP in Cos1 cells by fluorescence microscopy revealed that Shoc2-tRFP displayed a cytosolic distribution in serum-starved cells (Figure 1A). Shoc2-tRFP was also localized in the nucleus, and a small pool of the fusion protein was occasionally seen in intracellular vesicular structures. Upon activation of EGFR at 37°C, Shoc2-tRFP was found to be accumulated in the intracellular compartments (Figure 1B and C). Such vesicular accumulation of Shoc2-tRFP reached a maximum level within 12–15 min following EGF stimulation and was maintained for an additional 20–30 min. Shoc2-tRFP containing vesicles were situated mostly in the perinuclear area of the cells. Some vesicles were relatively static, while most of the vesicles showed rapid lateral and directed movement over short distances, characteristic of microtubule-dependent endosome motility (Movie S1).

To define Shoc2-tRFP containing vesicular compartments, Cos1 cells were transiently co-transfected with Shoc2-tRFP and either GFP-Rab5 or CFP-Rab7, resident proteins of early and late endosomes, respectively. Shoc2-tRFP was found in some CFP-Rab7 positive compartments (Figure 1B) and very rarely present in early endosomes labeled with GFP-Rab5 (Figure 1C). Shoc2-tRFP was not co-localized with internalized fluorescent transferrin, a marker of early and recycling endosomes (data not shown). These data suggest that Shoc2-tRFP containing compartments are likely to be late endosomes.

**Figure 2:**
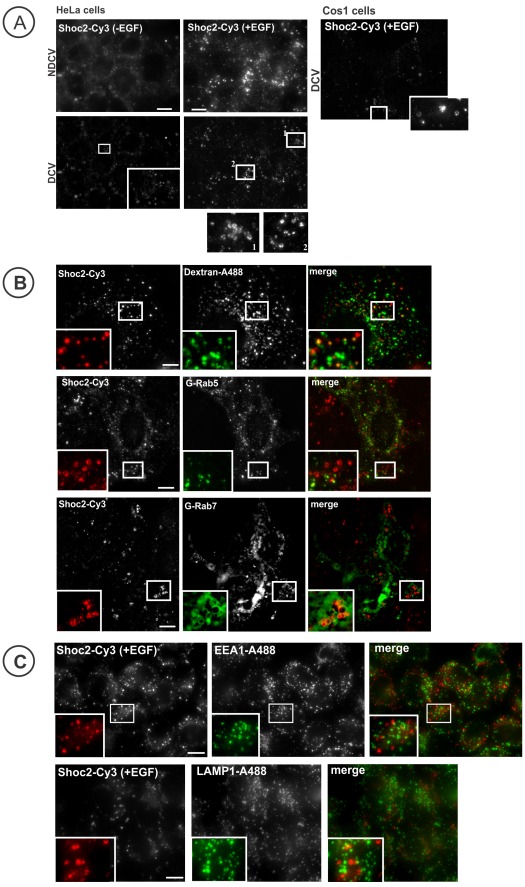
Localization of endogenous Shoc2. **A**, Serum-starved HeLa cells were treated (+EGF) or not (-EGF) with 10 ng/ml EGF for 12 min at 37°C. The cells were then fixed, permeabilized and stained with Shoc2 antibodies and secondary Cy3 donkey anti-rabbit antibodies. Images of HeLa cells before (NDCV) and after de-convolution (DCV) are shown. Deconvoluted Images for Cos1 cells are shown. Insets show high magnification images of the regions of the cell indicated by white rectangles. Scale bar, 10 µm. **B**, Serum-starved HeLa cells were incubated with 2 mg/ml Dextran-Alexa488™ for 2 hours and treated with EGF as in (***A***). **Below**, cells were transfected with either GFP-Rab5 or GFP-Rab7, treated as in *(* ***A*** *)* and then fixed, permeabilized and stained with antibodies to Shoc2 followed by secondary Cy3-conjugated donkey anti-rabbit antibodies. Insets show high magnification images of the regions of the cell indicated by white rectangles. 31.7±8.5% (SD) of Shoc2 immunoreactivity was colocalized with Dextran-Alexa488™. Scale bar, 10 µm. **C**, Serum-starved HeLa cells were treated as in *(* ***A*** *),* fixed, permeabilized and stained with the Shoc2 antibody combined with either EEA.1 or LAMP1 antibody. Secondary Cy3-conjugated donkey anti-rabbit and Alexa488-conjugated donkey anti-mouse antibodies were used. Insets show high magnification images of the regions of the cell indicated by white rectangles. Scale bars, 10 µm.

To confirm the finding of Shoc2 localization on endosomes, the distribution of endogenous Shoc2 was examined by immunofluorescence microscopy. In serum-starved HeLa or Cos1 (not shown) cells Shoc2 was found diffusely distributed throughout the cell (Figure 2A). EGF treatment resulted in accumulation of a pool of Shoc2 in intracellular vesicular compartments in both cell types (Figure 2A). The accumulation of endogenous Shoc2 in intracellular vesicles was more pronounced in HeLa cells, and therefore, majority of the following immunofluorescence experiments was carried in these cells. As in experiments in living cells, the subcellular distribution and shapes of individual Shoc2 containing structures were characteristic of endosomes or lysosomes. Hence, to examine the nature of Shoc2-containing compartments, the endosomal system of the cells was loaded with Dextran-A488™, and the cells were then treated with EGF. Significant co-localization of Shoc2 and Dextran-A488™ was observed in EGF-treated cells. Co-localization analysis of deconvoluted images revealed that approximately 32% of cellular Shoc2 was located in dextran-containing endosomes (Figure 2B), indicating that Shoc2 compartments are indeed of an endosomal origin. The same extent of colocalization of Shoc2-containing vesicles was observed with GFP-Rab7 positive endosomes, suggesting that Shoc2 translocate to a sub-population of late endosomes. Interestingly, Shoc2 immunoreactivity was clustered along the perimeter of large Rab7-endosomes (Figure 2B). Virtually no co-localization of Shoc2 with markers of early endosomes (EEA.1, Rab5) and lysosomes (LAMP1) was observed (Figure 2B and C), suggesting that endogenous Shoc2 does not translocate to these compartments. Thus, Shoc2 compartments could be a specialized population of late endosomes.

**Figure 3:**
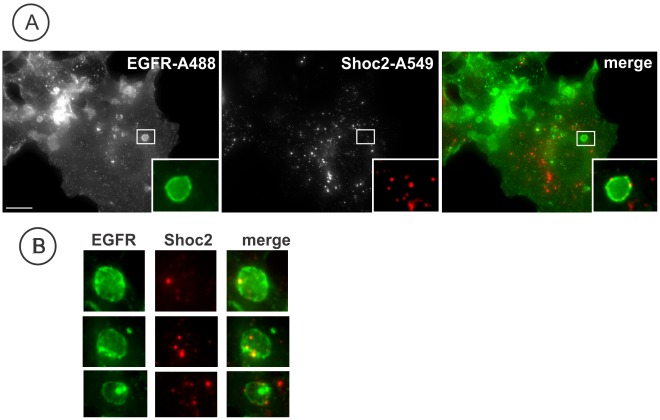
Endogenous Shoc2 localizes with active EGFR. **A**, Serum-starved Cos1 cells were treated with 10 ng/ml of EGF for 12 min at 37°C, fixed, permeabilized and stained with Shoc2 and EGFR (Ab528) antibodies followed by secondary Alexa548 donkey anti-rabbit and Alexa488 donkey anti-mouse antibodies were used. Insets show high magnification images of the regions of the cell indicated by white rectangles. Scale bars, 10 µm. **B**, High magnification images of the regions similar to those presented in *(* ***A*** *)* with examples of co-localization of Shoc2 and EGFR. Filter channels used for imaging of living cells as in *(* ***A*** *)* insets. Scale bars, 5 µm.

To examine whether Shoc2 accumulated in endosomes containing internalized EGFR, we used COS-1 cells that express a relatively high level of endogenous EGFR and have large endosomes, which facilitates light microscopic analysis. Cos1 cells were stimulated with EGF, fixed and co-stained with EGFR and Shoc2 antibodies. While most of Shoc2 endosomes did not contain EGFR, a pool of Shoc2 was co-localized with endosomal EGFR (Figure 3A). As in the case of Rab7 endosomes in HeLa cells (Figure 2B), clusters of Shoc2 were often located in the membrane of large endosomes (likely multi-vesicular bodies) containing EGFR (Figure 3A and B). Taken together, the data in Figures 1, 2, and 3 suggest that a subset of multi-vesicular bodies and late endosomes is the main site of Shoc2 localization in EGF-stimulated cells.

### RAS Activity and Clathrin are Necessary for Shoc2 Localization to Endosomes

**Figure 4:**
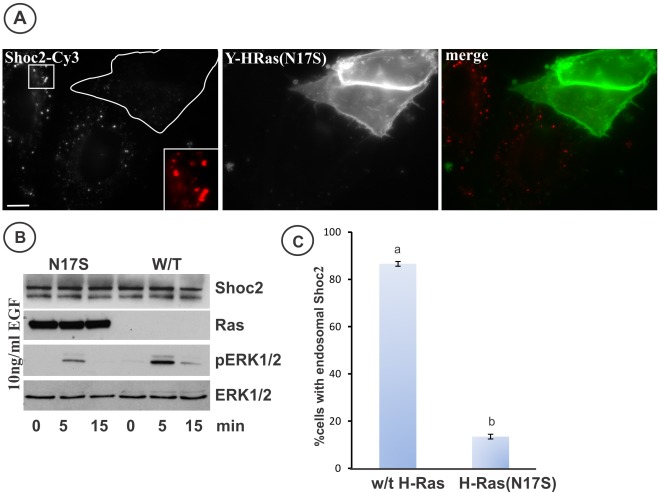
YFP-H-RAS (N17S) mutant inhibits Shoc2 recruitment to endosomes. **A**, HeLa cells were transfected with YFP-H-RAS (N17S), serum-starved and then incubated with 10 ng/ml EGF for 12 min at 37°C. Cells were then fixed, permeabilized and stained with anti- Shoc2 antibodies and secondary Cy3 donkey anti-rabbit. Insets show high magnification images of the regions of the cell indicated by the white rectangle. Cells expressing YFP-H-RAS (N17S) are outlined in the Shoc2 image. Scale bars, 10 µm. **B**, HeLa cells were transfected with the YFP-H-RAS (N17S) mutant and serum-starved. Cells were then incubated with 10 ng/ml EGF for 12 min at 37°C and lysed. The lysates were probed for Shoc2, RAS, phospho-ERK1/2 and total ERK1/2 by western blotting. **C**, Multiple images from the experiments exemplified in ***A*** were inspected, and the percentage of cells containing Shoc2 endosomes was calculated (+/−S.D.). The data are representative of 3 independent experiments, a vs. b, P<0.001 (one-way ANOVA test using SigmaStat 3.5 was used to determine differences).

To dissect the mechanisms of Shoc2 targeting to endosomes we tested whether activity of RAS, the key interacting partner of Shoc2, is necessary for the endosomal recruitment of Shoc2-tRFP. To this end, HeLa cells were transfected with YFP-H-RAS (S17N), a GDP bound mutant of RAS. This mutant is thought to have a dominant negative effect on the activity of RAS by occupying GTP-exchange factors of RAS [23], thus inhibiting EGF-induced, RAS-mediated ERK1/2 activation (Figure 4B). Cells expressing and not expressing YFP-H-RAS (S17N) were treated with EGF at 37°C for 10 min and then stained with Shoc2 antibodies. Figure 4A and C show that recruitment of Shoc2 to endosomes was impaired in cells overexpressing YFP-H-RAS (S17N) (outlined cells in upper panel of Figure 4A). In cells that did not express or expressed low levels of YFP-H-RAS (S17N) recruitment of Shoc2 to endosomes was intact or slightly reduced. These results suggest that RAS activity is required for Shoc2 endosomal localization.

**Figure 5:**
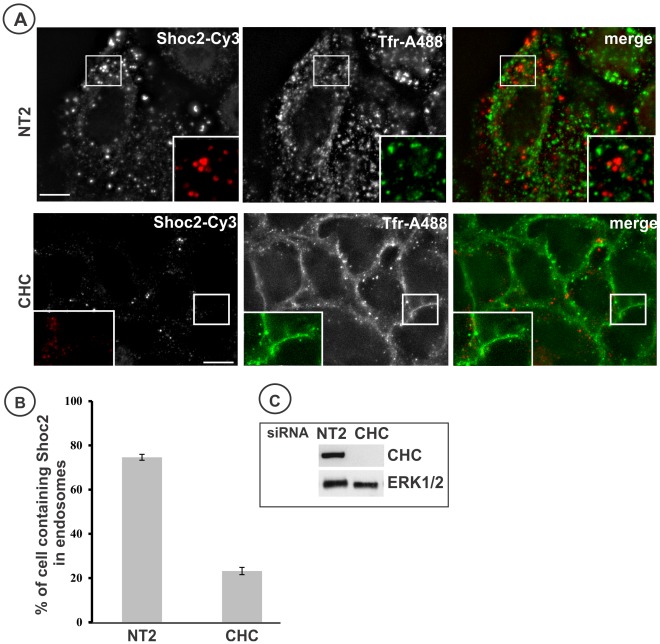
CHC siRNA inhibits Shoc2 recruitment to endosomes. **A**, HeLa cells were transfected with CHC or non-targeting (NT2) siRNAs and serum-starved. The cells were incubated with 2 ng/ml EGF and 5 µg/ml Tfr-TR for 10 min at 37°C, and fixed. Insets show high magnification images of the regions of the cell indicated by the white rectangle. Scale bar, 10 µm. **B**, Multiple images from the experiments exemplified in *(* ***A*** *)* were inspected, and the percentage of cells containing Shoc2 endosomes was calculated (+/−S.D.). The data are representative of 3 independent experiments. **C**, HeLa cells from the experiment in *(* ***A*** *)* were lysed and probed for CHC, and total ERK1/2 (loading control).

To investigate the role of clathrin-dependent endocytosis in Shoc2-tRFP targeting to endosomes, clathrin heavy chain (CHC) was depleted by siRNA. Knock-down of CHC with this duplex was previously demonstrated to efficiently block the endocytosis of EGF and transferrin [24]. The cells were stimulated with a low concentration of EGF (2 ng/ml), conditions favoring internalization of EGF-receptor complexes predominantly via clathrin coated pits. The blockade of endocytosis of transferrin labeled with Alexa488 (Tfn-A488) was used as control for the efficiency of CHC depletion in individual cells. As shown in Figure 5, CHC siRNA dramatically reduced targeting of Shoc2-tRFP to endosomes (Figure 5A and B). These data demonstrated that clathrin-dependent processes are involved in EGF-induced Shoc2-tRFP recruitment to endosomes.

### Shoc2 (S2G) Mutant is not Targeted to Late Endosomes

**Figure 6:**
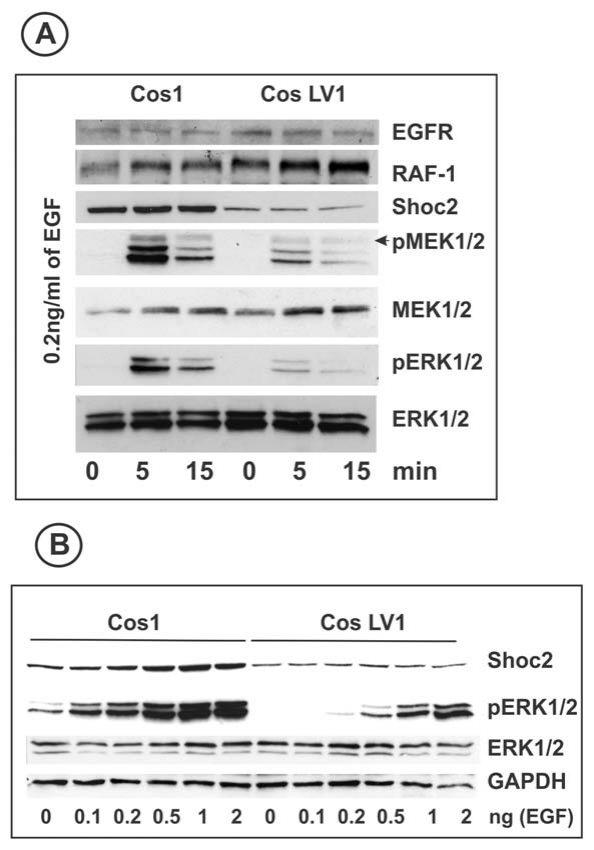
Shoc2 is required for ERK1/2 activation by EGF in Cos1 cells. **A**, Parental Cos1 and Cos1 cells stably expressing Shoc2-shRNA (Cos1-LV1) were serum-starved and treated with 0.2 ng/ml EGF for indicated times at 37°C. The lysates were probed for EGFR, Raf-1, Shoc2, activated ERK1/2 (pERK1/2), activated MEK1/2 (pMEK1/2), total ERK1/2 (ERK1/2) and MEK1/2 (MEK1/2). **B**, Parental Cos1 and Cos1-LV1 cells were serum-starved and treated or not (0) with increasing concentrations of EGF (0.1, 0.2, 0,5, 1, 2 ng/ml) for 12 min at 37°C. The lysates were probed for Shoc2, activated ERK1/2 (pERK1/2), total ERK1/2 and GAPDH (loading control).

Heterologous overexpression of the components of the ERK activation pathway and, in particular, scaffold proteins, in the presence of their endogenous counterparts often leads to the formation of non-specific complexes and sequesters binding partners from specific protein-protein interactions [9]. Therefore, to perform structure-function analysis of the Shoc2 role in EGFR signaling to ERK1/2, we used an RNAi approach. First, the most efficient siRNA sequence among 4 siRNA duplexes targeting Shoc2 (duplex #1) was identified (data not shown). Depletion of Shoc2 using this siRNA duplex significantly reduced ERK1/2 activity in EGF-treated cells (Figure S2). The effect of Shoc2 knockdown was most evident when the cells were stimulated with a low (0.2 ng/ml) concentration of EGF (Figure S2). Next, Cos1 cells with constitutive knock-down of Shoc2 (Cos1-LV1) were generated using lentiviruses carrying shRNA that was prepared based on the siRNA duplex #1 sequence. In order to prevent clonal variations due to the different sites of viral genome incorporation, a pool population of shRNA-expressing Cos-LV1 cells was used in subsequent experiments. Figure 6A shows that constitutive depletion of Shoc2 protein resulted in the dramatic decrease in the extent of phosphorylation of MEK1/2 and ERK1/2 upon EGFR activation. As expected, the effect of Shoc2 knockdown was most evident when the cells were stimulated with low (0.1–0.5 ng/ml) concentrations of EGF (Figure 6B). Such EGF concentrations are detected in human plasma and most tissues where EGFR is accessible to EGF [25], [26]. Transient expression of the Shoc2-tRFP mutant, in which 6 “silent” mutations were introduced to render it to be resistant to duplex #1 without changing its amino acid sequence (Shoc2-tRFP*), in Cos-LV1 cells has rescued EGF-induced ERK1/2 phosphorylation (Figure 6A). The ERK1/2 phosphorylation signal in these cells was lower than in parental COS1 cells, presumably, due to the fact that not all COS-LV1 cells expressed Shoc2-tRFP*.

**Figure 7:**
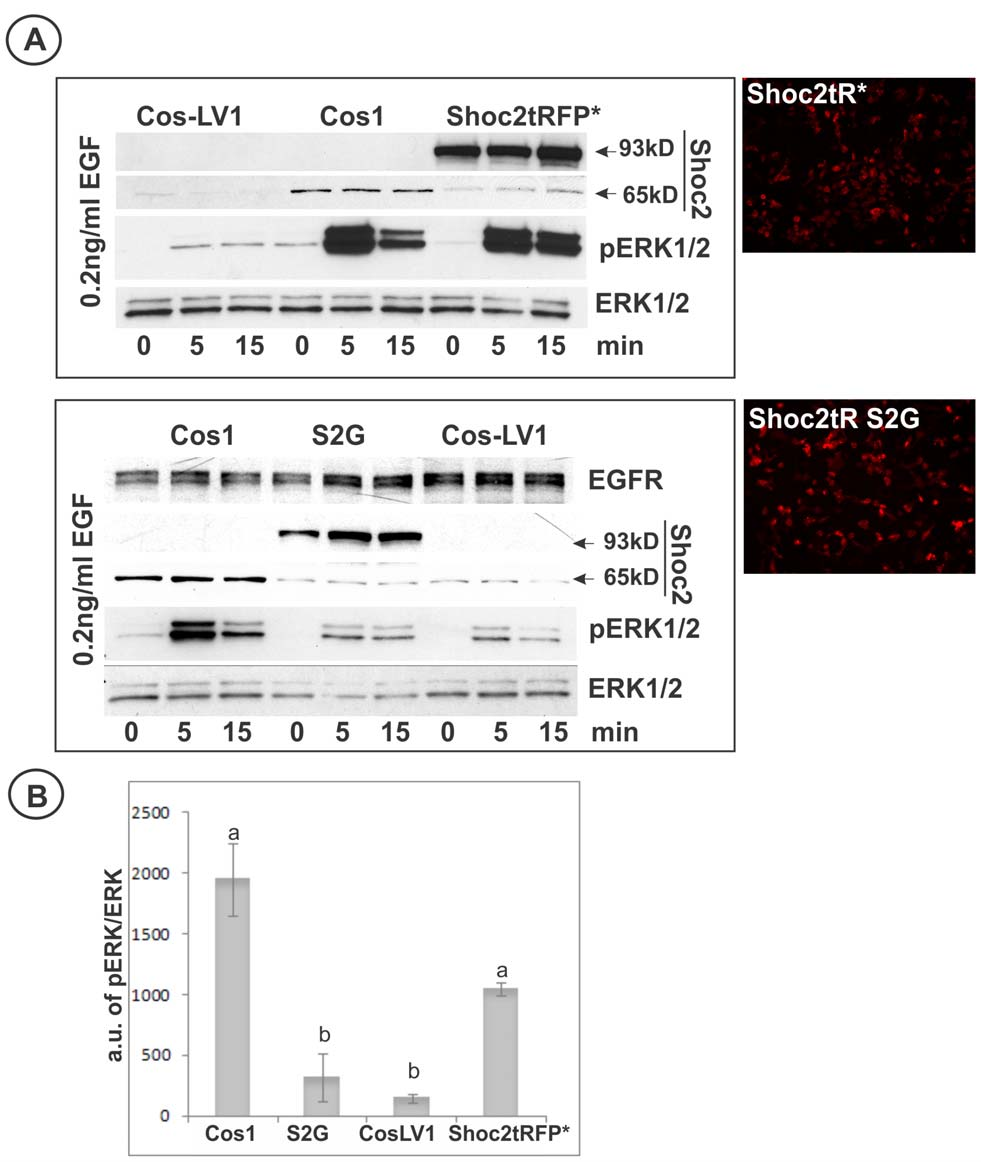
Wild-type Shoc2 but not Shoc2 (S2G) mutant rescues Shoc2 knockdown. **A**, Cos-LV1 cells were transiently transfected with full-length Shoc2-tRFP or Shoc2-tRFP (S2G) mutant. Cells were serum-starved and treated with 0.2 ng/ml EGF for indicated times at 37°C. The lysates were probed by western blotting for activated ERK1/2 (pERK1/2) and total ERK1/2 (ERK1/2). Low magnification images of Shoc2-tRFP* and Shoc2-tRFP* (S2G) presented to highlight expression efficiency of these proteins in Cos-LV1 cells. **B**, Multiple blots from the experiments exemplified in ***A*** were analyzed. Bars represent the mean values (±S.E., *n* = 3) of phosphorylated ERK1/2 activity normalized to total ERK in arbitrary units (pERK/ERK), a vs. b, P<0.05 (one-way ANOVA test using SigmaStat 3.5 was used to determine differences in phosphorylated ERK1/2 activity).

It has been proposed that overexpression of the Shoc2 mutant with the S2G substitution (serine 2 is mutated to glycine), found in Noonan-like syndrome patients, results in N-myristoylation and targeting of this Shoc2 mutant to the plasma membrane, and enhances EGFR-dependent ERK1/2 activity [22]. To test the effect of S2G mutation in the background of cells lacking endogenous Shoc2, the Shoc2-tRFP* (S2G) mutant was transiently expressed in Cos-LV1 cells (Figure 7A and B). At the expression levels that were maximally achievable for these constructs in Cos-LV1 cells, the Shoc2-tRFP* (S2G) mutant was unable to restore the ERK1/2 activity above the basal level of EGF-induced ERK1/2 activity in Shoc2-depleted cells (Figure 7A and B). These data suggest that the S2G mutation is inhibitory to the Shoc2 function in ERK activation.

**Figure 8:**
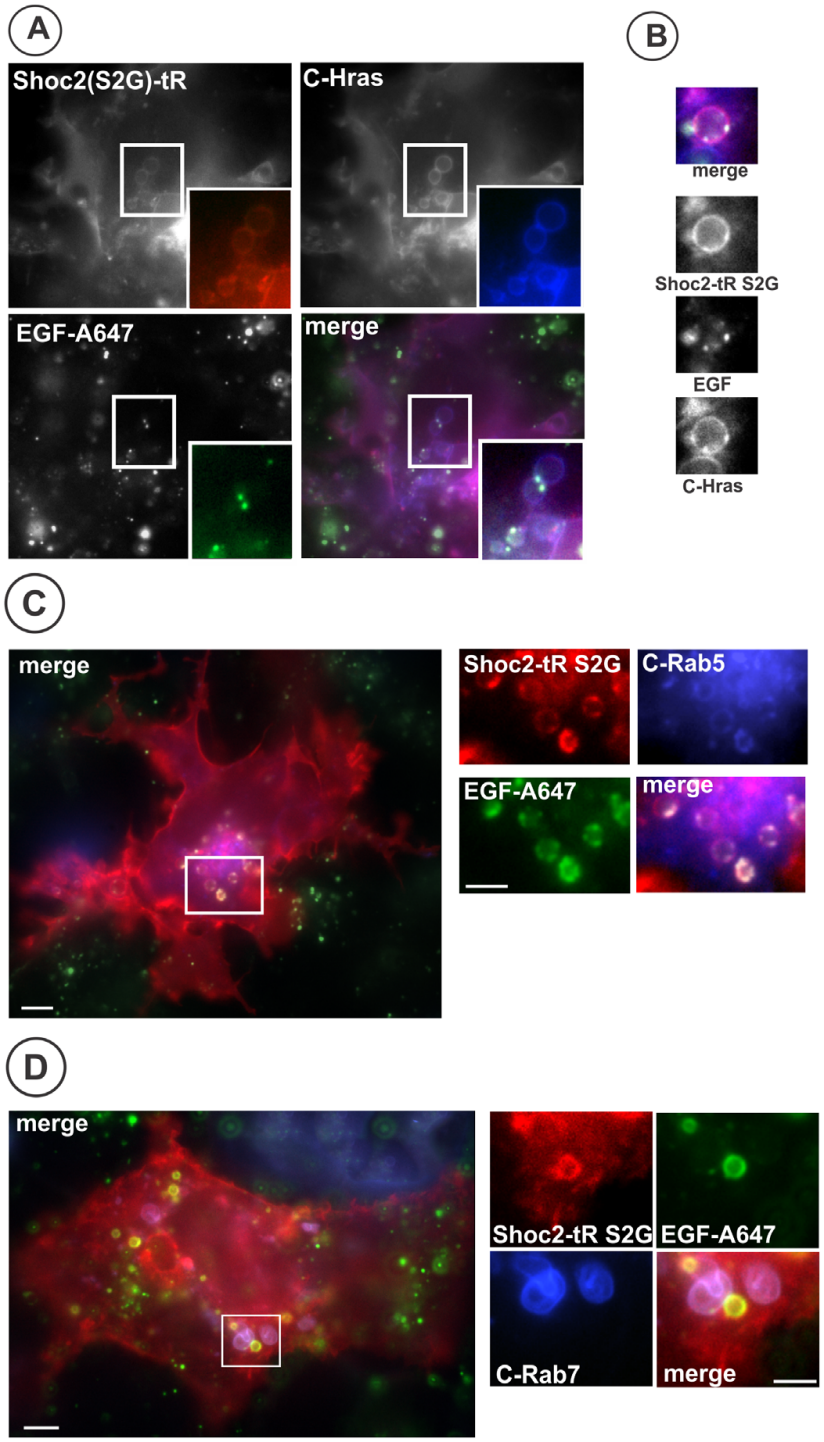
Shoc2 S2G mutant is co-localized with EGF, Rab5 and H-RAS. **A**, Cos1-LV1 cells were transiently transfected with Shoc2-tRFP* (S2G) mutant and CFP-H-RAS. Serum-starved cells were incubated with 10 ng/ml EGF-Alexa647 for 12 min at 37°C. Insets show high-magnification images of the regions of the cell indicated by white rectangles. Scale bar, 10 µm. **B**, High-magnification images of the regions similar to those that are presented in **A**-insets shown to highlight colocalization of the Shoc2 S2G mutant with EGF and H-RAS. **C**, Cos-LV1 cells were transiently transfected with Shoc2-tRFP* (S2G) mutant and CFP-Rab5. Serum-starved cells were treated with EGF-Alexa647 as in ***A***. Insets show high-magnification images of the regions of the cell indicated by white rectangle. Scale bar, 10 µm. **D**, Cos-LV1 cells were transiently transfected with Shoc2-tRFP* (S2G) mutant and CFP-Rab7. Serum-starved cells were treated with EGF-Alexa647 for 30 min. Insets show high-magnification images of the regions of the cell indicated by white rectangle. Scale bar, 10 µm.

Analysis of the subcellular localization of Shoc2-tRFP* (S2G) using live-cell fluorescence microscopy demonstrated that in serum-starved Cos1-LVI cells Shoc2-tRFP* (S2G) is located mainly in the plasma membrane (data not shown). It is likely that stable association of Shoc2-tRFP* (S2G) with the membrane is mediated by its N-myristoylation and interactions of the cluster of positively-charged amino acids in the amino-terminus of Shoc2 (residues 5–60). After treatment with EGF the mutant was accumulated in endosome-like compartments (Figure 8A, Movie S2). In contrast to endosomes containing wild-type Shoc2 (Figures 1, 2, and 3), the S2G mutant was highly co-localized with EGF-Alexa647 and YFP-H-RAS in endosomes (Figure 8A and B). Moreover, Shoc2-tRFP* (S2G) mutant was found to be well co-localized with endosomes containing CFP-Rab5, which was especially evident on “donut-shape” profiles of large endosomes (Figure 8C). This pattern of localization in the plasma membrane and endosomes is reminiscent of the subcellular distribution of K-Ras that is modified by farnesylation (a fatty-acid modification similar to myristoylation) and has a positively-charged region in the proximity to the farnesylation site [27], [28]. Most important, unlike wild-type Shoc2, Shoc2-tRFP* (S2G) was not significantly co-localized with CFP-Rab7 (Figure 8D). These data demonstrated that upon EGFR activation, Shoc2-tRFP* (S2G) is recruited to early endosomal compartments and that Shoc2 located on the plasma membrane and early endosomes is incapable of promoting ERK1/2 activation under conditions of cell stimulation with low EGF concentrations.

## Discussion

Assembly of macromolecular signaling complexes in intracellular membrane compartments, such as endosomes, Golgi apparatus and endoplasmic reticulum, and its importance in orchestrating the network of signaling processes are well established [3], [29], [30], [31], [32], [33]. Herein, we demonstrated endosomal localization of Shoc2, a putative scaffold protein that is important for signaling along the RAS-RAF-MEK-ERK cascade [19], [34]. Our immunofluorescence and live-cell imaging analyses showed that upon EGFR activation endogenous Shoc2 and transfected Shoc2-tRFP translocate to endosomes (Figures 1 and 2). Cordeddu and co-workers have previously reported that heterologously expressed and endogenous Shoc2 are present in the cytoplasm and nucleus, and that EGF stimulation causes translocation of cytosolic Shoc2 to membranes [22]. In our experiments, a pool of Shoc2-tRFP was also found in cell nuclei regardless of EGF stimulation (Figure 1). However, we have not detected a significant nuclear pool of endogenous Shoc2 (Figure 2). It is possible that differences in immunofluorescence staining methods and cell types are accountable for different patterns of Shoc2 localization observed in our experiments and by Cordeddu and co-workers [22]. On the other hand, consistent with the present study, the increased membrane pool of Shoc2 in EGF-stimulated cells [22] could result from the recruitment of cytosolic Shoc2 to endosomes, given that Shoc2 was not detected in the plasma membrane. Hence, we propose that endosomes are the main site of EGF-dependent targeting of Shoc2 in cells used in our study. The nature of these endosomal compartments is not fully understood. Many of the Shoc2-containing vesicles accumulated non-specific endocytic cargo like dextran, although only a small pool of Shoc2 was detected on endosomes containing internalized EGFR (Figure 3). Moreover, Shoc2 was not detected in Rab5/EEA.1 containing early endosomes where most activated EGFRs were located, suggesting that Shoc2 is not associated with the classical “signaling” endosomes [29], [35]. The highest extent of co-localization of the vesicle-associated Shoc2 was with Rab7, and thus these Shoc2 compartments likely represent a subset of late endosomes.

As clathrin knock-down inhibited endosomal translocation of Shoc2 (Figure 5), the mechanism of EGF-induced Shoc2 targeting to late endosomes involves clathrin-mediated endocytosis of either EGFR or another protein that anchors Shoc2 to the endosomal membrane. Shoc2 recruitment to endosomes was also decreased dramatically in cells overexpressing dominant-negative H-RAS (N17S) (GDP-bound) mutant suggesting that Shoc2 targeting to endosomes is downstream of RAS activation (Figure 4). Since GTP-binding of RAS leads to membrane translocation of RAF and activation of its kinase, efficient Shoc2 relocation to endosomes may require RAF activity. Shoc2 was demonstrated to be a part of the PP1C enzymatic complex activity, that removes the inhibitory phosphate from Ser259 of RAF in response to the growth factor stimulation, thus allowing activation of RAF kinase [21]. Interestingly, recently published data suggest that dephosphorylation of RAF S259 is the primary pathogenic mechanism in the activation of several RAF1 mutants identified in patients with Noonan syndrome [36], which emphasizes the role of the PP1C holoenzyme complex and Shoc2, particularly, in the regulation of ERK1/2 pathway. Therefore, it is tempting to assume that endosomal targeting of Shoc2 can be a part of the positive feedback regulatory loop necessary for proper RAF activation.

To examine the role of Shoc2 translocation to late endosomes in ERK activation, we took advantage of the observation that a single amino acid change (S2G) results in N-myristoylation and aberrant targeting of Shoc2 (S2G) mutant to the plasma membrane [22] and, in cells treated with EGF, to early (Rab5-positive, Rab7-negative) endosomes (Figure 8). To analyze Shoc2 function during signal transduction from EGFR using this Shoc2 mutant, we used an approach that involves the functional rescue of a shRNA-depleted endogenous Shoc2 by its tagRFP-fused wild-type or mutant version. Surprisingly, the S2G mutant did not rescue EGFR-mediated activation of the ERK1/2 pathway in Cos1 cells with constitutively depleted Shoc2, suggesting that S2G mutation inhibits Shoc2 function (Figure 7). These results appear to be inconsistent with the model proposed by Cordeddu and co-workers [22] in which S2G is considered to be a gain-of-function mutation. This model was based on the observation of a slightly stronger effect of overexpressed Shoc2 (S2G) mutant on ERK1/2 activity in Neuro2A cells as compared with the effects of overexpressed wild-type Shoc2. However, several considerations may explain apparent inconsistencies of the data, and suggest that, at least in some experimental model systems, the S2G mutation is inhibitory to Shoc2 function during EGF-induced signaling to ERK1/2.

Firstly, RNAi studies by others [20], [21] and our experiments (Figures 6 and 7) demonstrated that Shoc2 has significant role in EGF-induced ERK1/2 activation only when mammalian cultured cells, such as HeLa, HEK293 and Cos1, are stimulated with low, physiological concentrations of EGF (<1 ng/ml). Overexpression of wild-type or mutant Shoc2 increased the constitutive ERK1/2 activity, whereas the EGF-induced ERK1/2 activity was not changed [22]. This effect was observed in Neuro2A but not in HEK293 and Cos1 cells. Secondly, genetic analysis of vulval development in *C. elegans* demonstrated that SOC-2 (S2G) expression did not rescue SOC-2 mutation (*soc-2, ku167*). However, it is unclear whether the perturbed development in the presence of S2G mutation is the result of increased RAF activation in the vulva [22]. The effects of the S2G mutant may, therefore, be additionally attributed to mechanisms unrelated to the ERK activation pathway [22]. It should also be pointed out that not all mutations associated with the Noonan syndrome and clinically related disorders have a gain-of-function effect, and in particular, Noonan-like syndrome patients with Shoc2 (S2G) mutations manifest defects consistent with the reduced cell proliferation [37]. Thirdly, another level of complexity that can explain cell-dependent differences in the contribution of Shoc2 in ERK regulation is the existence of multiple forms of RAF that are activated and regulated by different and complex mechanisms. As an example, inhibition of the constitutively-active mutant of B-RAF by vemurafenib may lead to the compensatory up-regulation of signaling pathways leading to activation of c-RAF [38]. Interestingly, targeting of another signaling protein MEK2 to late endosomes required A-RAF and c-RAF activity but was not supported by constitutively-active B-RAF [33]. Finally, discrepancies in the data can be related to the differences in the experimental design. Our experimental model (Cos-LV1 cells) is reminiscent of the Noonan-like syndrome because Shoc2-S2G mutant was expressed in these cells in the absence of wild type Shoc2 (Figures 6 and 7). In experiments with Neuro2A cells, the S2G mutant was expressed in the presence of endogenous Shoc2 [22].

While further experimentation is necessary to reconcile all the data described above, a hypothetic model of spatial regulation of Shoc2 function and the effects of S2G mutation can be proposed whereby Shoc2 is important for signaling from EGFR to ERK1/2 if rate-limiting interactions of participating signaling components are not saturated (low EGF concentrations). Under these conditions mis-targeting of Shoc2 due to the S2G mutation eliminates cytosolic and late-endosomal pools of Shoc2, and results in dramatic inhibition of ERK activation. Therefore, either cytosolic or late-endosomal, or both of these pools of Shoc2 are necessary for ensuring an effective signal transduction from activated EGFR to ERK. More specifically, we hypothesize that a pool of Shoc2 that is targeted to Rab7-positive endosomes is important for EGF-dependent ERK1/2 activation because this endosomal targeting occurs in an EGF and Ras-dependent manner. In contrast, the cytosolic Shoc2 pool is decreased upon EGF treatment.

On the other hand, ERK activity supported by EGFR-independent signaling pathways or in the presence of high EGF concentrations (30 ng/ml) [22] can be enhanced by overexpression of the Shoc2 (S2G) mutant, probably, through the increase in the amount of Shoc2-RAS-RAF complexes at the plasma membrane and early endosomes. When expressed at very high levels (that are typically achieved in cells like Cos1 and HEK293) the Shoc2 (S2G) mutant may also increase signaling from the plasma membrane but additionally have a trans-inhibitory effect by preventing binding of Shoc2 interactors to the endogenous Shoc2 located in the cytosol and on late endosomes. We predict that, as in the case of stimulation of cells with high concentrations of EGF (Figure 6), constitutive activity of ERK1/2 would not be affected in Neuro2A and other cells by siRNA depletion of Shoc2.

To conclude, the critical role of Shoc2 during signaling to ERK1/2 under physiological conditions of EGFR activation is striking. Further detailed analysis of Shoc2 interactions and subcellular dynamics is in progress to generate a comprehensive model of Shoc2 function.

## Materials and Methods

### Reagents and Antibodies

EGF was obtained from Collaborative Research (Bedford, MA). EGF conjugated to Alexa fluor 647 streptavidin (EGF-Alexa647) and human Transferrin conjugated to Texas Red (Tfr-TR) were purchased from Molecular Probes (Eugene, OR); antibodies to EGFR, RAF-1, MEK1/2, ERK1/2, phospho-ERK1/2, phospho-MEK1/2, GAPDH were from Cell Signaling Technology; clathrin heavy chain antibodies (TD1) were from the American Type Culture Collection (ATCC); Shoc2 antibodies were from Abcam (USA); HA antibodies were from Covance. Pfu polymerase was purchased from Stratagene (La Jolla, CA).

### Expression Plasmids

The full-length human Shoc2 in pcDNA3.1 was kindly provided by Dr. Rodriguez-Viciana (UCL Cancer Institute, UK). To generate the tagRFP (red fluorescent protein) [39] (courtesy of Dr. V.V. Verkhusha, Albert Einstein College of Medicine) tagged version of Shoc2 a forward primer containing a *XhoI* site and reverse primer containing an *BamHI* site after the stop codon were used to amplify the human Shoc2 sequence by PCR. CFP/GFP-tagged Rab7 and Rab5 plasmids were described previously [40]. YFP-RAS (N17S) was kindly provided by Mark Dell’Acqua (University of Colorado Denver). 3xHA-MRAS and 3xHA-H-RAS were purchased from Missouri S&T cDNA Resource Center (www.cdna.org).

Four individual duplexes to human Shoc2 were obtained from Dharmacon (Lafayette, CO) and used for transient transfection. Clathrin Heavy Chain specific siRNA oligos were obtained from Dharmacon (Lafayette, CO) and were described previously [40]. To generate a plasmid stably expressing Shoc2 specific shRNA, pLVTHM vector was used (Addgene plasmid 12247). A set of oligonucleotides corresponding to the Shoc2 specific sequence of siRNA duplex #1 (5′GAAGAGAAUUCAAUGCGUU 3′) containing *MluI* and *ClaI* restriction sites were synthesized. The following primers were used:

5′CGCGTCCCCGAAGAGAATTCAATGCGTTTTCAAGAGAAACGCATTGAATTCTCTTCTTTTTGGAAT 3′, and 5′CGATTTCCAAAAAGAAGAGAATTCAATGCGTTTCTCTTGAAAACGCATTGAATTCTCTTCGGGGA 3′. Oligonucletides were annealed as described in [41] and ligated into pLV-THM1 using *MluI* and *ClaI* restriction sites. The pLVTHM-Shoc2 construct was verified by dideoxynucleotide sequencing. Point mutations in the Shoc2-tRFP construct were introduced using a QuickChange site-directed mutagenesis kit according to the manufacturer’s directions (Stratagene). Silent mutations in Shoc2-tRFP changed the DNA sequence but not the amino acid sequence, making the constructs resistant to shRNA knockdown. The following primers were used: 5′GAGCTCAACAAATGCCGGGAGGAAAACAGCATGAGGCTGGACTTATCCAAGAGAT 3′, and 5′ATCTCTTGGATAAGTCCAGCCTCATGCTGTTTTCCTCCCGGCATTTGTTGAGCTC 3′. The constructs were verified by dideoxynucleotide sequencing.

### Cell Culture and DNA Transfections

The human cervical carcinoma HeLa cells from ATCC, the human embryonal kidney 293FT cells from Invitrogen, and Cos1 from ATCC cells were grown in Dulbecco Modified Eagle’s Medium (DMEM) containing 10% fetal bovine serum (FBS) supplemented with Sodium Pyruvate, MEM-NEAA, Penicillin, Streptomycin, and L-Glutamate (Invitrogen). The transfections of DNA constructs were performed using Lipofectamine2000 (Invitrogen) and *Trans*IT® (Mirus Bio LLC) reagents. Expression of tagRFP-fused proteins was confirmed by Western blotting as described below.

### siRNA Transfections

To silence protein expression by RNA interference, HeLa and Cos1 cells were seeded in 12-well plates (50–60% confluent; 1 ml of DMEM/FBS per well) at least 20 hours before transfection. siRNA transfections were performed at 24–36 hour intervals according to the manufacturer's recommendations, using Dharmafect reagent 1 (Dharmacon). Three to four days post-transfection, the confluent cells were trypsinized and half of the cells were seeded on glass-bottom dishes (MatTek, MA) for fluorescence microscopy analysis, while the other half were used for Western blot analysis. The cells were then incubated in serum-free and phenol red free medium containing 0.2% bovine serum albumin (BSA) for 20 hours prior to the microscopy experiments. The efficiency of the siRNA knockdown was validated by Western blotting.

### Immunoprecipitation and Western Blot Analysis

The 293FT cells grown in 35-mm dishes were placed on ice and washed with CMF-PBS, and the proteins were solubilized in Triton X-100/glycerol/HEPES lysis buffer supplemented with 100 mm NaCl, and protease inhibitors for 20 min at 4°C [42]. Lysates were then centrifuged at 16,000×*g* for 20 min to remove the insoluble material. Lysates were incubated with appropriate antibodies for 2 h and the immuno-complexes were precipitated using Protein A or G Sepharose. Immunoprecipitates and aliquots of cell lysates were denatured in the sample buffer at 95°C, resolved by electrophoresis, and probed by Western blotting with various antibodies followed by the chemiluminescence detection. Western blotting was done as described previously [43]. Several x-ray films were analyzed to determine the linear range of the chemiluminescence signals, and the quantifications were performed using densitometry analysis mode of the QuantityOne software (Bio-Rad, Inc).

### Immunofluorescence Staining and Analysis

Cells grown on glass-bottom dishes were either treated or not treated with 10 ng/ml of EGF and washed with Ca^2+^, Mg^2+^ -free phosphate buffered saline (CMF-PBS). The cells were then fixed with freshly prepared 4% paraformaldehyde (Electron Microscopy Sciences, Hatfield, PA) for 10 min at room temperature and permeabilized using 0.5% Tween for 20 minutes at room temperature. Immunostaining was performed according to manufacturer’s recommendations for the antibodies used. For dextran uptake experiments, HeLa cells were pulse-labeled in serum-free DMEM with 2 mg/ml dextran-Alexa488 (10,000 MW, lysine fixable (Invitrogen)) for 2 h at 37°C in 5% CO_2_, followed by a PBS wash and fixation in 4% paraformaldehyde (Electron Microscopy Sciences, Hatfield, PA). All images were acquired using a Mariannas Imaging system consisting of a Zeiss inverted microscope equipped with a cooled CCD CoolSnap HQ (Roper, CA), dual filter wheels and a Xenon 175 W light source, all controlled by SlideBook software (Intelligent Imaging Innovations, Denver, CO). The detection of Alexa488 fluorescence was performed using a FITC filter channel, Alexa549 fluorescence using an mRFP or Cy3 channel; Alexa647– using a CY5 channel. Images were acquired using 2×2 binning mode. Image analysis was performed using the SlideBook 5 software. Co-localization analysis was performed using the co-localization statistical module in SlideBook 5 software.

### Fluorescence Imaging of Living Cells

The cells were re-plated 24 hours before the experiment onto 35-mm glass-bottom dishes and kept in serum free and phenol red free medium containing 0.2% BSA for 16–20 hours. The cells were imaged at room temperature using a Mariannas™ workstation. The detection of GFP fluorescence was performed using a FITC filter channel, tRFP fluorescence using a Cy3 channel; Alexa647– using a CY5 channel, CFP using a CFP channel. Images were acquired using 2×2 binning mode.

To quantify the endosomal localization of Shoc2, several three-dimensional images were acquired under each experimental condition. The cells were categorized based on visual inspection into two groups: (i) cells containing at least one endosome decorated by tRFP and (ii) cells containing no tRFP-decorated endosomes. The number of cells containing endosomal Shoc2-tRFP was expressed as a percent of total cells analyzed.

## Supporting Information

Figure S1 **Shoc2 binding of RAS in Cos1 cells.** 293FT cells were transiently co-transfected with expression vectors encoding tagRFP-tagged Shoc2 or its D175N mutant, and either 3xHA-MRAS or 3xHA-HRAS. 48 h post-transfection, cells were harvested, and cell lysates were subjected to immunoprecipitation with anti-HA antibody as described under “Materials and Methods”. The entire bound fraction (IP) was analyzed by immunoblotting with Shoc2 antibodies to detect Shoc2 and HA antibodies to detect Ras. Cell lysates (Input) were immunoblotted with anti-HA antibody to monitor expression of Ras proteins or Shoc2 Abs to monitor expression of Shoc2 and corresponding mutant used in panel IP. Results in each panel are representative of three independent experiments.
(TIF)

Figure S2 **Shoc2 is required for ERK1/2 activation by EGF in Cos1 cells.** Cos1 cells were transiently transfected with Shoc2 specific siRNA duplex #1 (Shoc2) or non-targeting siRNA (NT). Shoc2 was detected in cell lysates using Shoc2 antibodies. Cells were starved and then incubated without (−) or with 0.2 or 10 ng/ml EGF (+) for 5 and 15 min at 37°C. The lysates were probed for activated ERK1/2 (pERK1/2) and total ERK1/2 (loading control).
(TIF)

Movie S1 **Cos1 cells were transfected with Shoc2-tRFP and then treated with 10 ng/ml of EGF for 10 min at 37°C (as described in the** **Fig. 1B** **).** Time-lapse images were acquired every 5 sec during 2 min at room temperature.
(MOV)

Movie S2 **Cos-LV1 cells were transfected with Shoc2 (S2G)-tRFP, CFP-Rab5 and then treated with 1 ng/ml of EGF-Alexa647 for 10 min at 37°C (as described in** **Fig. 1B** **).** Time-lapse images were acquired every 30 sec during 12 min at room temperature.
(MOV)
