# Supplementary figures and images for: Shoc2 Is Targeted to Late Endosomes and Required for Erk1/2 Activation in EGF-Stimulated Cells
Source: PLoS One. 2012 May 14;7(5):e36469. doi: 10.1371/journal.pone.0036469 (PMC3351432; doi:10.1371/journal.pone.0036469)

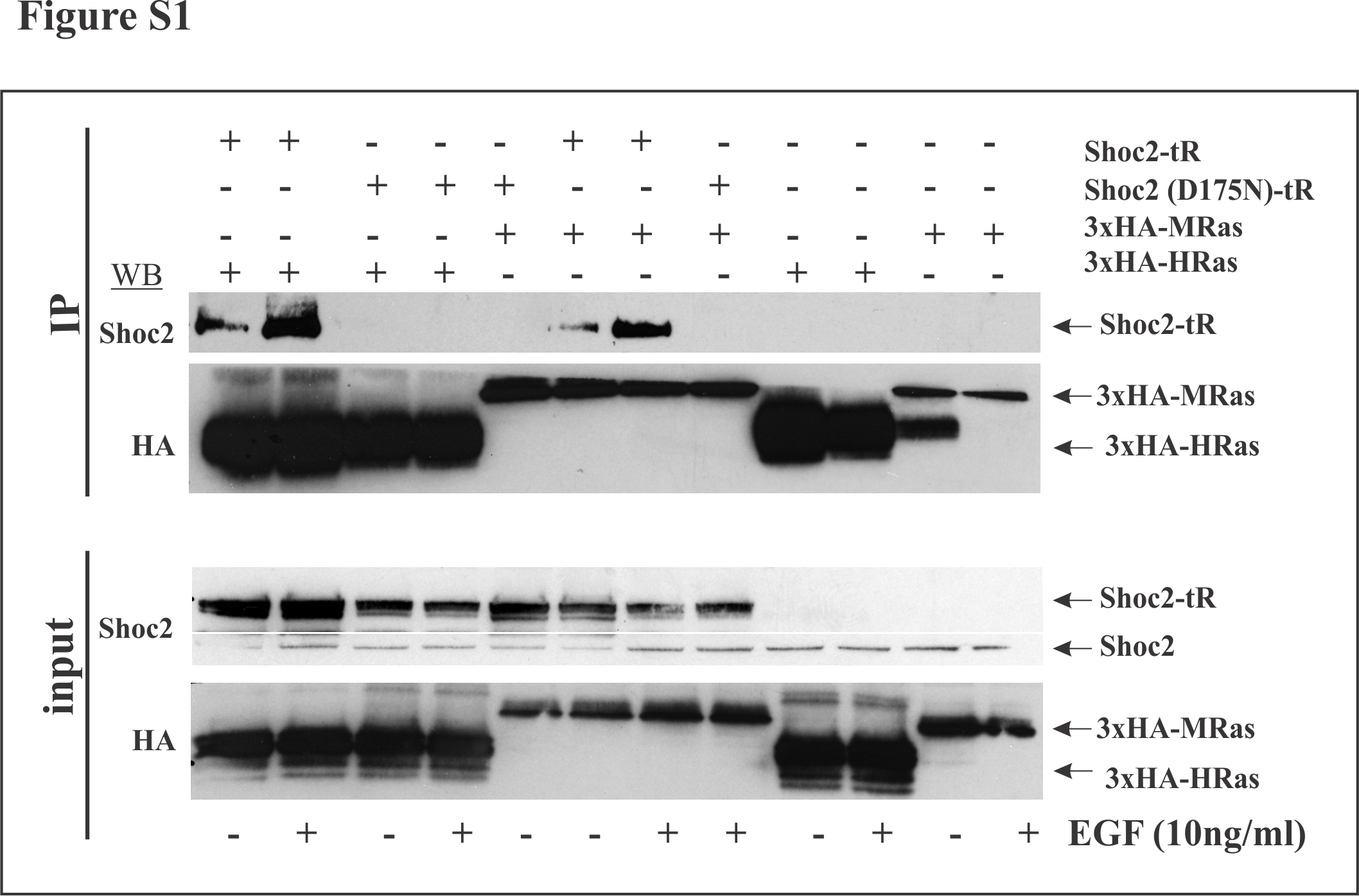

Supplement: Figure S1 — Shoc2 binding of RAS in Cos1 cells. 293FT cells were transiently co-transfected with expression vectors encoding tagRFP-tagged Shoc2 or its D175N mutant, and either 3xHA-MRAS or 3xHA-HRAS. 48 h post-transfection, cells were harvested, and cell lysates were subjected to immunoprecipitation with anti-HA antibody as described under “Materials and Methods”. The entire bound fraction (IP) was analyzed by immunoblotting with Shoc2 antibodies to detect Shoc2 and HA antibodies to detect Ras. Cell lysates (Input) were immunoblotted with anti-HA antibody to monitor expression of Ras proteins or Shoc2 Abs to monitor expression of Shoc2 and corresponding mutant used in panel IP. Results in each panel are representative of three independent experiments. (TIF) [file pone.0036469.s001.tif]

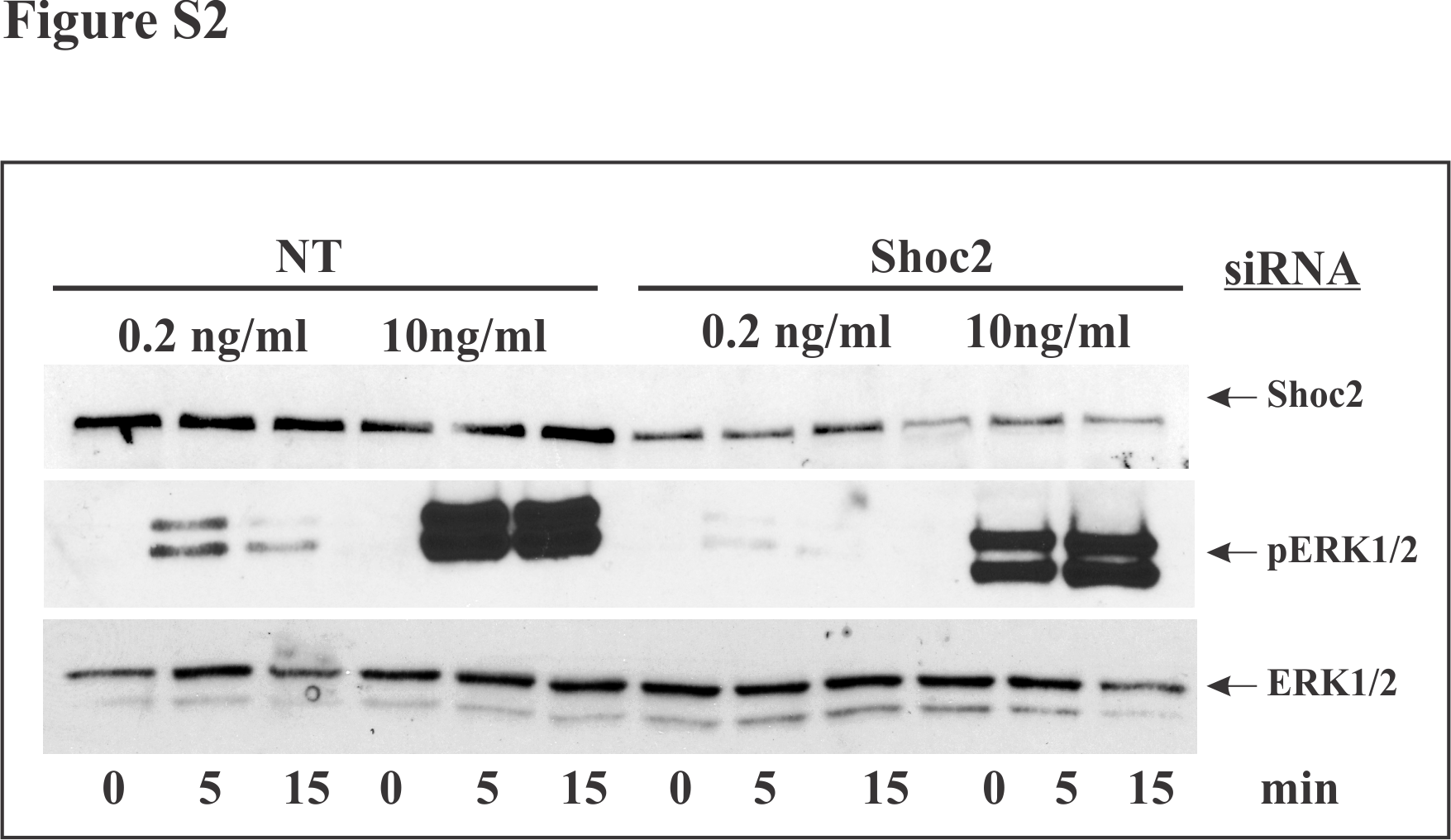

Supplement: Figure S2 — Shoc2 is required for ERK1/2 activation by EGF in Cos1 cells. Cos1 cells were transiently transfected with Shoc2 specific siRNA duplex #1 (Shoc2) or non-targeting siRNA (NT). Shoc2 was detected in cell lysates using Shoc2 antibodies. Cells were starved and then incubated without (−) or with 0.2 or 10 ng/ml EGF (+) for 5 and 15 min at 37°C. The lysates were probed for activated ERK1/2 (pERK1/2) and total ERK1/2 (loading control). (TIF) [file pone.0036469.s002.tif]
